# After the first wave: What effects did the COVID-19 measures have on regular care and how can general practitioners respond to this?

**DOI:** 10.1080/13814788.2020.1798156

**Published:** 2020-08-13

**Authors:** Henk van Weert

**Affiliations:** Department of General Practice, University of Amsterdam, Amsterdam, the Netherlands

## Introduction

Recently, a friend of mine talked about his 92-year-old mother who lived in a home for the elderly, which was locked down four months ago to prevent COVID-19 infections. During the lockdown, she was moved from her small apartment into a psychogeriatric care ward. Both her sons were not involved in the decision-making process, as they were not allowed to visit her. She changed from being a reasonably autonomous person into a depressed, incontinent and dependant person, without much hope for recovery.

A colleague told me about a 48-year-old man who visited his practice after several days of worsening eye pain and visual deterioration. The diagnosis was quite straightforward: keratitis. Subsequent consultation with an ophthalmologist confirmed the feared sequelae: the cornea was lost. When the patient was asked why he had waited so long before visiting, he answered that he had not wanted to bother his doctor, who was so busy in these times of COVID.

Worldwide, the COVID-19 pandemic continues to grow. On 1 July, the virus reached a sad milestone, with mortality exceeding half a million people. The USA, South America and the Middle East are now leading in (registered) mortality [1]. With the European COVID-19 pandemic receding, we can start to ‘calculate the bill’. This evaluation is necessary, because ‘winter is coming’ – to quote a well-known television series, and fear exists for a second wave of the pandemic. The medical ingredients for this bill are significant, including not only the effects of the pandemic and the costs of measures taken but also the unintended consequences of these actions and the sequelae arising from postponed care. We will have to learn lessons from the past months.

## Policy against COVID-19 and unwanted side effects

In daily healthcare, measures including social distancing and the use of facemasks and eye-protection seem reasonably effective, with a relative risk of transmission of the virus of between 0.2 and 0.4 [2]. However, problems of a lack of personal protective equipment (PPE) and inadequate distribution to the health care facilities caused significant concerns. Intensive care units (ICU), hospital wards and emergency care facilities were prioritised in many countries. Primary care, social care providers, retirement homes, and nursing homes were typically at the end of the queue despite their overwhelming needs. The virus struck hard in these settings, including residential home care and care facilities for the elderly. Many older people died, and facilities were completely locked down: no visitors were allowed for many months. At this moment, it is difficult to judge the results of that policy, but social isolation and segregation from beloved ones certainly are not salutary for our elderly. Unfortunately, my friend’s story was no rarity.

The impact of lockdown measures on patients with chronic illnesses is yet to be assessed. Limited data from Greece indicate an increase in distress and somatisation – but not in anxiety and depression, yet – during quarantine among patients with a chronic illness [3].

Because of fear for overcrowded ICU’s, hospitals partly closed down and reallocated their facilities and personnel to critical care settings to prepare for an invasion of acutely ill patients, who would require long-term therapy. Although about one-third of the ICU patients with COVID-19 die, this strategy proved reasonably successful and probably saved thousands of lives, at least in the short term.

In primary care, with a shortage of PPE, social distancing was recommended, and GPs (i.e. general practitioners, family physicians) started rationalising physical entrance to the practice, expanded telephone and email communications and quickly introduced video consultations [4]. As a result, patients were sometimes hesitant to ‘trouble’ their GP or postponed a visit because of fear of catching the virus. Even more worrying, the rationalisation of hospital services delayed diagnostic testing and evaluation of referred patients. GPs found it challenging to refer patients – which often failed.

## Missed care

This combination of measures culminated in an unprecedented decrease in health care services delivered. The WHO reported that in 92% out of 155 responding countries, staff was reassigned from non-COVID to COVID related services. That shift mainly affected rehabilitation and chronic care, but cancer treatment and acute cardiovascular care were also often involved [5]. Two examples illustrate this ‘hidden pandemic’. In Spain, the number of invasive procedures for acute myocardial infarction at the onset of the pandemic decreased by 40%, which not only resulted in death but also increased survivors’ disability [6]. In Greece, strict lockdown measures were implemented from mid-March, and the number of patients consulting with chest pain, dyspnoea or palpitations who visited a cardiology emergency centre decreased sharply. Although the fraction of consulting patients, admitted to the hospital increased, the absolute number of myocardial infarction patients sharply decreased [7]. Until now, there is no explanation for this hidden morbidity and – probably – mortality. Additional research is needed to explain the impact of the decrease in the use of emergency department services during the pandemic.

Although the provision of acute care might look impressive, other services, including oncology, may suffer. In Europe, every year, almost 4 million people are diagnosed with invasive cancer, more than 350,000 patients each month [8]. In the Netherlands, during the first three months of the epidemic, diagnosed cancer was about 75% of usual; and a month after the alleviation of the epidemic measures, it did not reach at the expected number. For skin cancer (excluding basal cell carcinoma), the reduction was 50% [9]. For some cancers, it might be less troublesome to be discovered a few months later, e.g. cancers diagnosed by population screening. However, in many cancers, timely detection should not be postponed too long in order to prevent diagnosis at a more advanced stage. Catching up, however, is a tremendous job, for which facilities are lacking. A reasonable estimation of postponed care is about 5000 new cancers not diagnosed (yet) compared to the previous year in the Netherlands. If the numbers in the Netherlands equal those in Europe, we are 245,000 cancers behind [10].

Moreover, as far as oncology is concerned, it is not only about new diagnoses. Postponed care for prevalent cancer will have serious consequences as well. In oncology, often operation and post- or preoperative treatment is involved. Operations have been postponed due to a reduction in surgery capacity in hospitals, and chemotherapy has been postponed because of its immunosuppressive side effects. Furthermore, the inclusion of new patents in trials has often been delayed.

Similar problems may have arisen in other life-threatening or highly debilitating diseases. In the Netherlands alone, the estimation is that compared to the previous year, about 2.5 million referrals from primary to secondary care have not been made. While some claim that this crisis is a welcome opportunity to get rid of unnecessary health care, it is highly unlikely that this applies to all 2.5 million referrals. These figures probably contain much hidden morbidity. In primary care, the effects of postponing care are not as visible yet. However, the decreased number of cancer diagnoses certainly has a relation with decreased numbers of referrals by GPs, as well as by postponing diagnostic evaluations in specialised care. Somehow, this ‘missed care’ will return to the health care system, which is still suffering from COVID-19. A shortage of anything will be our part if no appropriate action is taken.

## What could general practitioners do?

GPs could try to catch up on delayed care, try to keep their practices accessible during an outbreak or ‘second wave’, act in the chain of testing, tracing and quarantine, and participate in the evaluation of new technology and health care procedures.

To catch up on delayed care, GPs can draw up a list of patients they have not seen in a while. They can contact patients with chronic conditions and patients at high-risk for health problems, e.g. patients with mental illness and patients with difficult access to health care. The current crisis is adversely affecting the mental health of the population, and primary care will be the first to notice this. Primary care teams may identify these issues among their patients and provide appropriate integrated care.

In addition, GPs can review telemedicine consultations in recent months for possibly missed clues, overlooked laboratory results or missed referrals. GPs can sift through referrals and check whether patients have arrived at the right specialist and have been followed up correctly. In a patient – suspected of cancer or other serious illness – who is not evaluated appropriately, the GP should take action. These ‘catch-up’ activities may require additional personnel, which should be trained for this task.

To keep general practices accessible, the separation of COVID care from ‘regular’ care may need to be continued. In the event of the ‘second wave’, patients should not hesitate to consult their GP face-to-face [11]. Therefore, GPs could collaborate at a district or city level to concentrate COVID care in locations other than their regular practices. Furthermore, these collaborative organisations would be the appropriate level to engage with diagnostic services to develop diagnostic protocols (triage, testing) and to act as distributors for PPE for primary care and home care staff.

Testing, tracing, and quarantine are the essential public health measures in an epidemic. Germany is an excellent current example. In countries where public health services are less well developed, GPs might take responsibility for the implementation of this policy, at least within their practices.

GPs should engage in the evaluation of novel communication technologies like video consultation and other telemedicine applications, which undoubtedly provide solutions for a part of the problems, both in acute and in chronic care [12,13]. In this evaluation, the limitations of these new technologies must be considered. For example, we do not exactly know which information is lost compared to face-to-face consultations. Evaluation in terms of increase or decrease of correct diagnostic or prognostic interpretation is warranted. The sharp decrease in diagnosed malignancies of the skin in the Netherlands might – at least partly – be caused by the application of video instead of face-to-face consultations.

Another example is the extent to which telemedicine is an adequate way to provide health care to patients with mental or social problems. This group of patients may be most hesitant to visit their GP. Reports from Italy and Spain showed a marked increase in psychiatric problems like depression, anxiety, somatoform, and alcohol-related disorders during the last economic crisis [14]. When unemployment strikes, this will undoubtedly happen again [15].

GPs should also be involved in evaluating newly developed or revitalised diagnostic and prognostic tests to separate COVID-patients from other patients by telephone and to triage common symptoms. The second wave of COVID patients would form a threat to our healthcare systems, and we should better be safe than sorry.

Finally, we need to evaluate whether our policy to prioritise ICU and hospital care and to isolate our older patients have been the right choices. In the event of a second wave of the COVID-19 pandemic in Europe, we need to be better informed about the effects of such prioritisation. In view of their experiences in recent months, GPs and geriatric specialists are pre-eminently the disciplines that can make an expert contribution to this policy evaluation.
